# Recent Advances in Metal–Organic-Framework-Based Nanocarriers for Controllable Drug Delivery and Release

**DOI:** 10.3390/pharmaceutics14122790

**Published:** 2022-12-13

**Authors:** Ziao Zong, Guanghui Tian, Junli Wang, Chuanbin Fan, Fenglian Yang, Feng Guo

**Affiliations:** 1 School of Laboratory Medicine, Youjiang Medical University for Nationalities, Baise 533000, China; 2 Modern Industrial College of Biomedicine and Great Health, Youjiang Medical University for Nationalities, Baise 533000, China; 3 Reproductive Medicine, Guangxi Medical and Health Key Discipline Construction Project of the Affiliated Hospital of Youjiang Medical University for Nationalities, Baise 533000, China

**Keywords:** metal–organic framework, drug delivery, release, nanocarrier

## Abstract

Metal–organic frameworks (MOFs) have a good designability, a well-defined pore, stimulus responsiveness, a high surface area, and a controllable morphology. Up to now, various MOFs have been widely used as nanocarriers and have attracted lots of attention in the field of drug delivery and release because of their good biocompatibility and high-drug-loading capacity. Herein, we provide a comprehensive summary of MOF-based nanocarriers for drug delivery and release over the last five years. Meanwhile, some representative examples are highlighted in detail according to four categories, including the University of Oslo MOFs, Fe-MOFs, cyclodextrin MOFs, and other MOFs. Moreover, the opportunities and challenges of MOF-based smart delivery vehicles are discussed. We hope that this review will be helpful for researchers to understand the recent developments and challenges of MOF-based drug-delivery systems.

## 1. Introduction

It is important to effectively improve targeted drug delivery and controlled drug release to reduce the damage toward normal tissues in modern medical science. Recently, more attention has been focused on the development of nanotechnology, biomedicine, and nanocarriers for controllable drug delivery and release to enhance the therapeutic effects and reduce adverse reactions [1,2,3]. Among many factors, nanocarriers have emerged as an effective nanotechnology to load and release drugs [4]. Up to now, various functional materials have been prepared and used as nanocarriers to realize this goal, such as polymer [5], graphene [6], metal oxide [7], quantum dot [8], mesoporous silica [9], and so on. Although some functional nanoscale materials show good performance in the area of drug delivery, some shortcomings greatly limit their widespread applications, including high toxicity, low-load capacity, unsatisfactory biocompatibility, uncontrollable release, and poor controllability. Hence, the design and exploration of outstanding nanocarrier materials are significantly vital for controllable drug delivery and release. Notably, porous materials are potential candidates in this application domain because they have a high surface area, abundant pores, and an adjustable microenvironment to enhance the loading capacity and controllable drug release in the target lesion site [10,11,12,13,14,15]. The highly ordered crystalline porous material with an exact structure is an ideal platform to investigate the relationship between skeleton structures and drug delivery/release properties.

Recently, metal–organic frameworks (MOFs) have had good designability, well-defined pores, stimulus responsiveness, a large surface area, and a controllable morphology [16,17,18,19]. Various MOFs have been developed and utilized as functional materials in many applications, such as gas sorption [20,21,22,23,24,25], heterogeneous catalysts [26,27,28,29,30,31], luminescent sensors [32,33,34,35,36], electrochemical aptasensors [37,38,39,40,41], and in biomedical applications [42,43,44,45]. By virtue of the advantages of porous MOFs, they provide an available platform for highly efficient drug loading and controlled drug release in specific contexts. Nanosized MOFs with an adjustable morphology are conducive to drug delivery [46]. Notably, the biocompatibility of MOFs must be considered as a prerequisite for practical biological applications. Metal ions and organic ligands are the most common components of MOFs and remarkably affect the biocompatibility. Hence, nontoxic ligands (such as terephthalic acids, imidazoles, cyclodextrins, etc.) and metal ions (such as Fe^3+/2+^, Ca^2+^, Mg^2+^, etc.) have attracted more attention to construct MOFs in the application of drug delivery. Moreover, targeted drug release is an important consideration in clinical applications [47], which can be realized via different external stimulus responses such as light [48,49,50], the pH value [51,52,53], and temperature [54,55,56,57]. Compared with thermal- and light-stimulus responsibility, acid stimulation is applied more in MOF-based drug carriers for cancer treatment because of the weakly acidic environment of cancer cells and the fragile coordination bond. When there is no obvious difference between the sick part and other normal parts, thermal- and light-sensitive smart carriers are more conducive to achieving a good targeted therapeutic effect. According to these considerations, the rational design and synthesis of sensitive smart MOFs is becoming an increasingly important approach for targeted drugs. Up to now, porous MOFs have been used as potential nanocarriers for controllable drug delivery and release [58,59,60,61,62]. Some representative outstanding investigations have been reported in recent decades. For example, J. An and others prepared a porous anionic zinc–adeninate bio-MOF-1 as a functional material for cation-triggered drug release [63]. H. Su and coauthors reported a highly porous medi-MOF-1, constructed using Zn and curcumin, for ibuprofen (Ibu) delivery with good biodegradation and cytotoxicity [64]. Zheng etc. developed a one-pot approach to combine the synthesis of ZIFs and the anticancer drug doxorubicin (Dox) encapsulation in 2016 [65]. M.H. Teplensky and coworkers utilized a temperature-treatment approach to delay the drug release, resulting in a more efficacious therapy [66]. A chiral-Zn-based MOF was prepared as a drug delivery with a high-drug-loading amount and slow 5-fluorouracil (5-FU) release [67]. Since this field is still developing rapidly, more relevant investigations have been reported over the last five years. Hence, a timely summary given the prospects of the research in this field is of great significance.

**Scheme 1:**
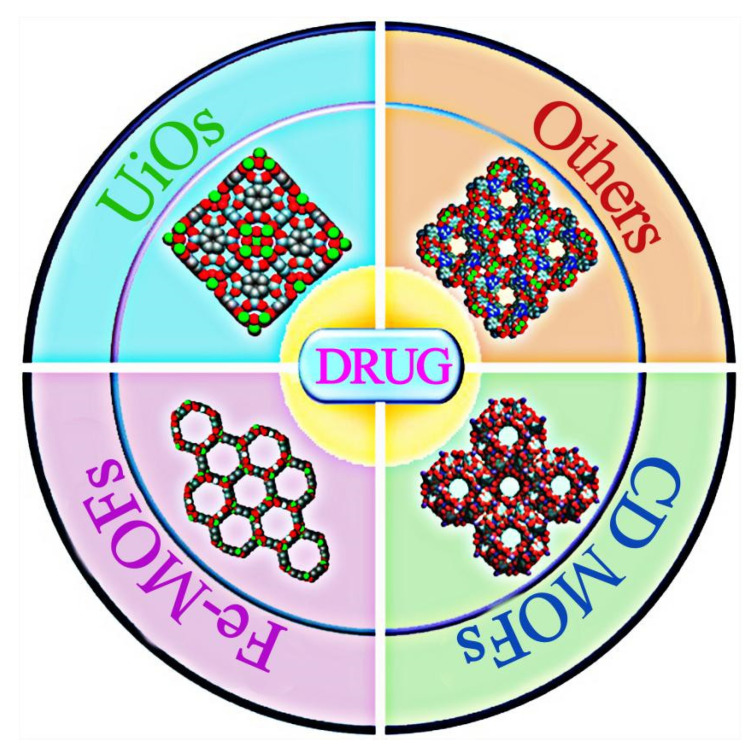
Schematic illustration of MOF-based nanocarriers for drug delivery and release.

Herein, we comprehensively summarize MOF-based nanocarriers for controllable drug delivery and release over the last five years. This review will be helpful for researchers to understand the recent developments and challenges of MOF-based drug-delivery systems. All reported MOFs in this field are divided into four categories (Scheme 1), including University of Oslo MOFs (UiOs), Fe-MOFs, cyclodextrin MOFs (CD MOFs), and other MOFs, because these MOFs not only have the advantages of MOFs but also possess a low toxicity and good biocompatibility. Meanwhile, some representative examples are discussed and highlighted in detail in this review (Table 1). Finally, we provide the opportunities and challenges of MOFs in drug-delivery and release applications.

## 2. Zr-Based UiOs

MOFs are constructed via the coordination assembly of metal ions/clusters and organic linkers. Generally, the stability of most MOFs is not enough to meet the needs of many actual applications because of the weak coordination bonds between the metal nodes and bridging linkers. In order to enhance the physical and chemical stability of MOFs, many approaches have been developed in recent years [90,91,92,93,94,95]. Notably, Zr-based UiOs, as one class of highly stable MOFs, were successfully designed and prepared via multinode Zr(IV) clusters and organic ligands with carboxylic acids such as UiO-66, UiO-67, and PCN-222 [96,97,98,99]. In addition, various functional groups can be easily introduced in MOFs, including -NO_2_, -NH_2_, -COOH, -SH, and -SO_3_H [100,101,102]. Moreover, the particle size of UiOs is well controlled via available approaches [103,104]. By virtue of the advantages of Zr-based UiOs, a lot of effort has been focused on the development of these MOFs for drug delivery and release.

**Figure 1:**
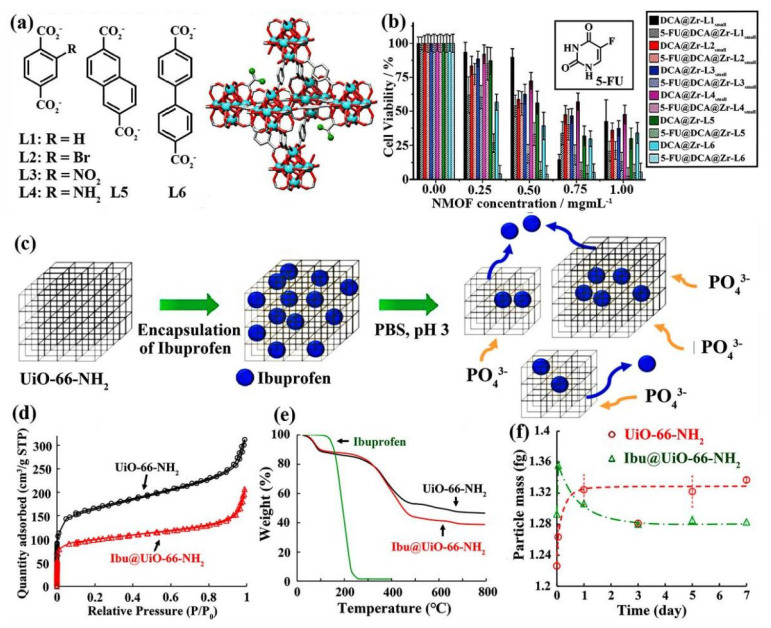
Defective Zr-MOFs: (**a**) Structure and (**b**) cell viability. Reproduced with permission from Ref. [68], copyright (2018) the Royal Society of Chemistry. (**c**) Preparation of Ibu@UiO-66-NH_2_ and its application. (**d**–**f**) Characterization and drug release of UiO-66-NH_2_ and Ibu@UiO-66-NH_2_. Reproduced with permission from Ref. [69], copyright (2019) the American Chemical Society.

The organic ligands and particle sizes of Zr-based UiOs are important factors that affect their properties of drug loading, biocompatibility, and toxicity. A typical example is reported by I. A. Lázaro and coauthors in 2018 (entry 1, Table 1) [68]. The authors skillfully chose dichloroacetic acid (DCA) as a modulator, which is a pyruvate D-kinase inhibitor and is overexpressed in cancer cells, to prepare a series of nanoscale Zr-MOFs using the coordination assembly of Zr_6_ clusters and different organic ligands, including 1,4-benzenedicarboxylate (bdc), bdc derivatives (-Br, -NO_2_, and -NH_2_), 2,6-napthalenedicarboxylate, and 4,4′-biphenyldicarboxylate (L1–6 in Figure 1a). Due to the introduced DCA and the strong coordination interaction between DCA and Zr(IV), the as-synthesized samples with many structural defects were well-dispersed nanoparticles (NPs) (Figure 1a). The powder X-ray diffraction (PXRD) patterns of the Zr-MOFs (L1–4) were obviously broad and weak peaks, illustrating that they were defective particles with a small size because of the partially substituted terephthalate linkers by the DCA molecules. The scanning electron microscopy (SEM) images illustrated the particle size of the DCA@Zr-L1_small_ was in the range of 10-30 nm, but the DCA@Zr-L5 NPs had a larger particle diameter (232 ± 30 nm). The N_2_ physisorption isotherms at 77 K under 1 atm of the DCA@Zr-L1_small_ exhibited an obvious hysteresis loop caused by defective structures. The structural characteristics of these MOFs make them potential carriers for drug delivery. Additionally, 5-FU is a common anticancer drug which was used as a drug mode to investigate the cytotoxicity of the as-synthesized 5-FU@DCA@MOFs and DCA@MOFs towards MCF-7 cells. It was found that the cell viability gradually reduced with the increasing MOF concentration, confirming the successful intracellular drug delivery in the MCF-7 cells. For smaller Zr-MOF precursors, the dose–response performance of the 5-FU@DCA@Zr-L1_small_ was more significant to decrease the cell viability to 21 ± 7% at 1 mg mL^−1^. The cell viability of the 5-FU@DCA@Zr-L5 and L6 obviously reduced to 7 ± 6% and 4 ± 6%, respectively (Figure 1b). Moreover, the 5-FU@DCA@MOFs showed better performance than the free 5-FU in the low-concentration range, which may be probably attributed to the synergistic effect of 5-FU and DCA, and the rapid cell absorption. This work developed an available approach to construct defective Zr-MOF NPs with good therapeutically efficiency. In addition, H.-L. Wang et al. prepared UiO-66-NH_2_ NPs to encapsulate Ibu in an acidic (pH ≈ 3) phosphate buffer solution (PBS) (Figure 1c, entry 1, Table 1) [69]. The N_2_ isotherm of the Ibu@MOF was lower than that of MOFs because of the successful encapsulation of Ibu in the MOFs (Figure 1d), which was consistent with the result of the thermogravimetric analysis (TGA) curves (Figure 1e). Particle masses of the UiO-66-NH_2_ and Ibu@UiO-66-NH_2_ firstly increased after soaking in PBS (pH ≈ 3) due to the adsorbed phosphates (PO_4_^3−^). Then, the particle mass of the Ibu@UiO-66-NH_2_ decreased to 1.307 ± 0.004 fg after 1 day, which was consistent with the decreased Ibu of 0.049 fg/Ibu@UiO-66-NH_2_ (Figure 1f). Another nanoscale-Zr-based MOF, namely, UiO-67-NH_2_, with an average particle size of 150 nm, was synthesized via the solvothermal method in the presence of triethylamine as a shape modifier (entry 3, Table 1) [70]. Camptothecin (CPT) is widely utilized as an anticancer drug. Nano-UiO-67-NH_2_ reached a maximum drug-loading amount of 36.53 wt% at a CPT concentration (300 mg L^−1^). Compared with the surface area of the UiO-67-NH_2_ (240.14 m^2^ g^−1^), CPT@nano-UiO-67-NH_2_ dramatically decreased to 83.12 m^2^ g^−1^ due to the immobilization of CPT in the nano-MOFs. The drug-delivery experiment illustrates that the CPT release process contains three stages on account of the released CPT molecules from the surface and channel of the MOF-based nanocarriers. The result illustrates that nanoscale Zr-MOFs are beneficial for controllable drug delivery and release using nano-Zr-MOFs-based nanocarriers. Meanwhile, other Zr-MOFs, including PCN-222 and UiO-66-COOH, have been prepared to deliver different drugs [105,106,107,108,109,110]. According to these investigations, functional groups are easily introduced in organic linkers to affect the particle size of MOFs, the cytotoxicity, and the drug-carrying capacity. Meanwhile, PO_4_^3−^ is a main ion in the biological liquid environment which can be considered as a regulator to destroy the skeleton and release drugs via the competitive coordination of PO_4_^3−^ and metal ions. To enhance the drug-treatment effect, the rational design of nanoscale porous Zr-MOFs with a low-toxic ligand can effectively load and perform the controlled release of drugs in real biological systems.

**Figure 2:**
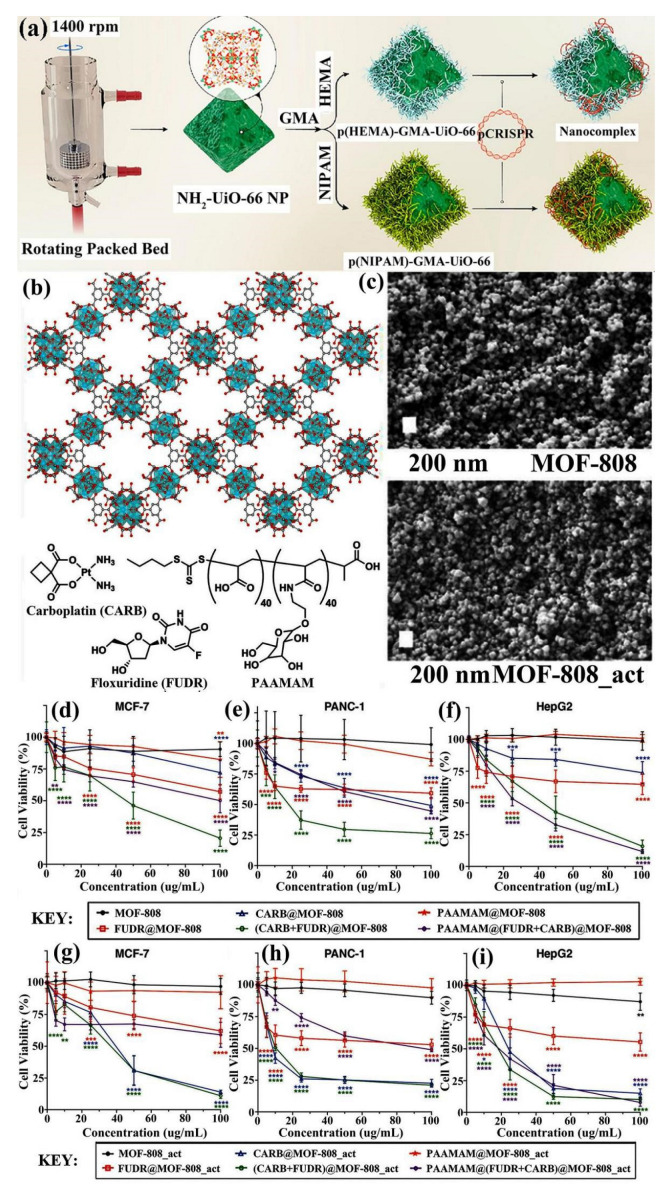
(**a**) Polymer-coated MOFs with pCRISPR. Reproduced with permission from Ref. [71], copyright (2021) the American Chemical Society. (**b**) Structure and morphology of MOF-808 and MOF-808_act. (**c**) Scanning electron micrographs of the nanoparticulate samples (scale bars 200 nm). (**d**–**i**) Cell viability. Reproduced with permission from Ref. [72] copyright© 2022 The Authors. Published by the American Chemical Society. (*p* ≤ 0.01 (**), *p* ≤ 0.001 (***), and *p* ≤ 0.0001 (****)).

Anticancer therapy always requires different drugs, so it is a meaningful work to develop one MOF carrier to simultaneously load multiple anticancer drugs. In 2020, R. S. Forgan’s group synthesized a defective Zr-MOF (UiO-66) via a multivariate modulation approach. The prepared sample could load three drugs to significantly enhance the selective anticancer cytotoxicity [111]. Subsequently, Rabiee et al. synthesized NH_2_-UiO-66 NPs, which are further coated by two different polymers, namely, *p*(HEMA) and *p*(NIPAM), based on 2-hydroxy ethyl methacrylate (HEMA) and *N*-isopropylacrylamide (NIPAM) organic monomers (entry 4, Table 1) [71]. The polymer-coated NH_2_-UiO-66 NPs, including *p*(HEMA)-GMA-MOF and *p*(NIPAM)-GMA-MOF, had high-Dox-load efficiencies of 51.4 and 55.9%, respectively. Then, plasmid (p)CRISPR was tagged on the surface of the polymer-GMA-MOFs to obtain the drug/gene dual-delivery systems (Figure 2a). This work developed a promising platform for the drug/gene delivery in biomedical applications. Another representative work is reported by F. D. Duman and cooperators in 2022 (entry 5, Table 1) [72]. The authors facilely synthesized MOF-808 NPs by the coordination interaction of Zr_6_ clusters and 1,3,5-benzenetricarboxylate linkers using acetic acid as a modulator. The MOF-808 NPs can simultaneously deliver two drugs, including floxuridine (FUDR) and chemotherapeutic agents carboplatin (CARB), which are further modified by a poly(acrylic acidmannose acrylamide) (PAAMAM) glycopolymer coating (Figure 2b). The activated sample was denoted as MOF-808_act to compare with the original MOF-808. As seen in Figure 2c, both samples of MOF-808 and MOF-808_act are octahedral NPs with a particle diameter of ~100 nm. The cytotoxicity of the MOF, PAAMAM, and PAAMAM-coated MOF toward MCF-7, PANC-1, and HepG2 cells at 72 h were investigated at different initial concentrations (Figure 2d–i). It was found that the individual composite without the drug had no obvious cytotoxicity, with a cell viability value above 80%. The drug-loaded MOF-based composites can decrease considerably the cell viability, confirming that MOFs not only improve the therapeutic performance of individual CARB and FUDR toward cancer cells, but also exhibit a synergistic effect of both drugs in the cancer treatment. These examples provide an available platform to utilize porous MOF NPs as nanocarriers for multidrug loading. However, it is still necessary to develop more effective MOF NPs to load diversified drugs for improving the synergistic treatment effect. Notably, it is a great challenge to control the appropriate proportion of different drugs in MOFs due to different sizes, solubility, and the host–guest interaction of drugs and MOFs. One option that could be considered is to use MOFs with a hierarchical pore structure to carry different drugs.

**Figure 3:**
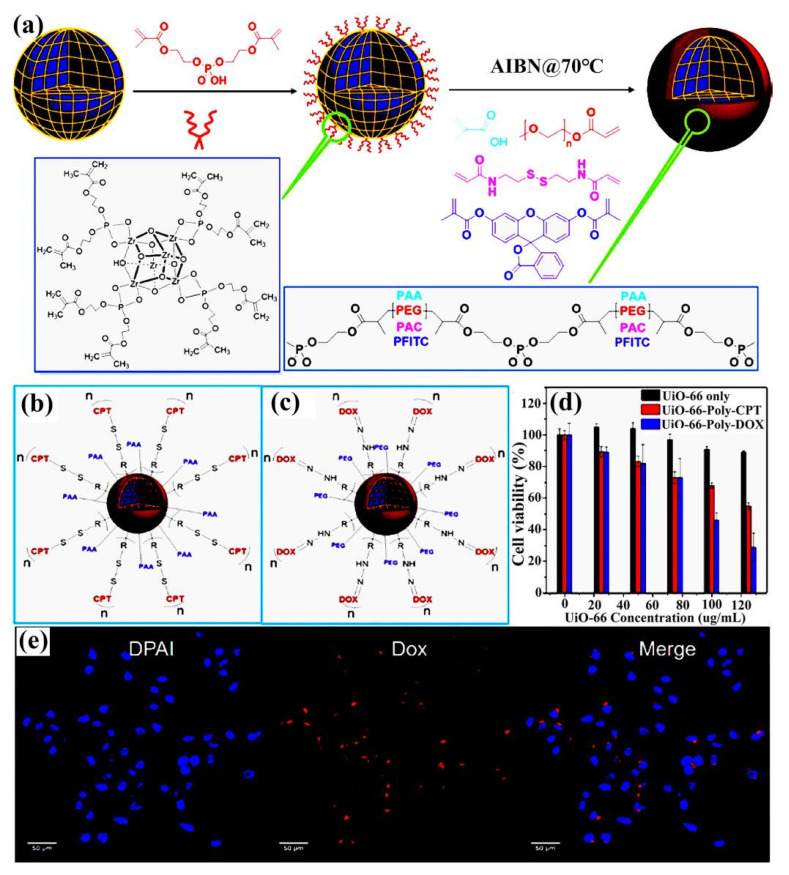
(**a**) Polymer-coated MOFs. (**b**) GSH- and (**c**) acidic pH-responsive drug-delivery systems. (**d**) Cytotoxicity of different materials. (**e**) Confocal imaging of the cancer cell with UiO-66-poly-Dox. Reproduced with permission from Ref. [73], copyright (2019) Wiley-VCH.

Sensitive smart MOFs have attracted more attention in cancer therapy through the targeted identification of drug delivery [112]. Targeted drug delivery not only increases the effective use of drugs and the therapeutic effect, but also reduces the damage toward normal cells. Notably, the poor stability of most MOFs in phosphate-based media biological applications is because of the strong coordination interaction between phosphate and metal ions. To overcome this intractable problem, Y. Liu and coauthors rationally developed polymer-wrapped MOFs via a feasible in situ polymerization strategy to improve the physicochemical stability in physiological conditions by virtue of the surface protective action of polymers covered on MOF NPs. Meanwhile, the polymer-modified MOFs also exhibited a stimulus-responsive intracellular drug release (entry 6, Table 1) [73]. As depicted in Figure 3a, *bis*[2-(methacryloyloxy)ethyl] phosphate (BMAP) molecules are anchored on the external surface of the nanoscale MOFs via the coordination interaction between metal ions and phosphate functional groups, which are further polymerized by different organic monomers in the presence of azobisisobutyronitrile (AIBN) to generate polymer coatings on the MOFs (Figure 3b). Polyethylene glycol is used to mix with organic monomers to copolymerize on the surface of MOF NPs to enhance the aqueous solubility. The prodrug camptothecin (CPT) breaks away from UiO-66 to kill cancer cells (Figure 3d). The other hydrazine monomer is copolymerized with polyethylene glycol to cover nanoscale UiO-66 particles (Figure 3c). The Dox can be cleaved from Dox-conjugated MOFs under the acidic environment of the cancer cell to achieve targeted drug therapy (Figure 3d). Especially, the MOF vehicle with dual drugs of CPT and Dox has excellent killing efficiency toward cancer cells. The confocal imaging in Figure 3e confirms the successful Dox delivery toward U87MG cancer cells via UiO-66-poly-Dox. This work opens up a novel approach to neatly unify the highly physiological stability and stimulus-responsive drug release of MOF-based nanocarriers. Another acid-sensitive nanoscale UiO-66 was utilized as a pulmonary drug delivery carrier to evaluate the effect of the defect caused by the loss of the ligand in the drug-delivery application [113]. The result demonstrates that nanoscale Zr-MOFs with adjustable properties are considered as a potential platform for high-efficiency pulmonary drug delivery with a good biocompatibility. Compared with normal cells, most cancer cells present a weak acidic environment. Hence, acid-sensitive smart materials or functional groups can be considered as a switch to control the drug release encased in MOFs.

**Figure 4:**
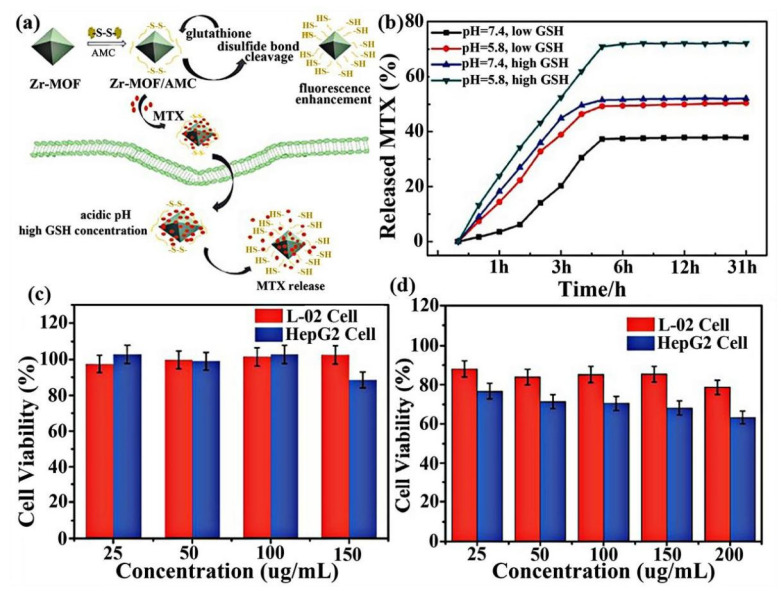
(**a**) The Zr-MOF/AMC/MTX nanocarrier and (**b**) its MTX release. Cell viability of (**c**) Zr-MOF/AMC and (**d**) Zr-MOF/AMC/MTX in L-02 and HepG2 cells. Reproduced with permission from Ref. [74], copyright (2020) Royal Society of Chemistry.

Furthermore, C. Lin et al. synthesized a Zr-MOF with acetaldehyde-modified-cystine (AMC) to generate Zr-MOF/AMC, which can not only determine the glutathione (GSH) concentration for cancer diagnosis but also exhibits pH/GSH dual-responsive methotrexate (MTX) release in the field of cancer treatment (Figure 4a, entry 7, Table 1) [74]. In comparison with the SEM and EDX elemental mapping of Zr-MOF, Zr-MOF/AMC has the S element from AMC in the EDX elemental mapping, suggesting the successful preparation of the Zr-MOF/AMC composite. The MTX release experiments show that the released MTX amount of Zr-MOF/AMC at the pH value of 7.4 was lower than that under a solution with a pH of 5.8 with the same concentration of GSH, which was mainly attributed to the partial hydrolysis and fracture of the –C=N bond in AMC (Figure 4b). Under the same pH condition, the MTX release effect from nanocarriers at 20 mM GSH was significantly higher than that at a low GSH concentration (5 mM), because the high GSH concentration can cleave the –S–S– bond of AMC and destroy the skeleton structure of the MOFs. Different concentrations of Zr-MOF/AMC and MTX-loaded Zr-MOF/AMC were incubated in L-02 and HepG2 cells (Figure 4c,d). The cell viability was over 80% for both cells with Zr-MOF/AMC at 150 mg mL^−1^, confirming the good histocompatibility and low toxicity of Zr-MOF/AMC. The survival rate of the L-02 cell was still above 80% after incubation with Zr-MOF/AMC/MTX, confirming the low released amount of MTX in normal cells. In contrast, only a 63% survival rate of the HepG2 cell was found in the presence of Zr-MOF/AMC/MTX at 200 mg mL^−1^. The results illustrate that the pH/GSH stimuli-responsive nanocarrier can perform a controlled drug release in cancer cells with a low toxicity toward normal cells. HepG2 cells showed a stronger green fluorescence than that of *N*-ethylmaleimide (NEM)-treated HepG2 cells, suggesting that the selectively visualized detection of GSH in living cells can be accomplished using Zr-MOF/AMC. Hence, the Zr-MOF/AMC composite is a potential nanocarrier for the pH/GSH dual-responsive drug delivery and offers a functional material for discriminating cancer cells. Another selenium-containing polymer-encapsulated Zr-MOF was prepared as a photoinduced vehicle to release Dox for the combination of photodynamic therapy and chemotherapy [114]. To realize the target drug delivery and release, sensitive smart MOF-based carriers can be rationally customized according to the unique application environment. Some effective strategies have been developed to achieve this goal, such as the reasonable choice of metal ions/clusters, organic linkers, and stimulus-responsive species. Hence, the exploration of more sensitive MOF-based nanocarriers is an important field for targeted drug delivery and reduced side effects.

According to these reported nanoscale Zr-MOFs in the drug-delivery application, appropriate MOF carriers should possess a large surface area, a small size, a sensitive stimulus response, and a fine biocompatibility. Hence, smart MOFs or their hybrid-materials-based nanocarriers can be controlled, designed, and constructed to enhance the drug-treatment effect. The stimuli-responsive component was assembled with MOFs to achieve the targeted drug delivery and decrease the side effects. Multidrugs were successfully loaded into MOFs via various interactions, such as the coordination bond, hydrogen bond, and physical absorption.

## 3. Fe-MOFs

In order to enhance the biocompatibility and reduce the toxicity toward normal cells, the choice of metal cation is one of the most important factors. As we know, bioactive metal cations, such as Mg^2+^, Ca^2+^, and Fe^2+/3+^, have been used to react with organic linkers to synthesize biocompatible MOFs [115,116,117,118,119,120], which provides a useful platform for controlled drug delivery and release. Fe is a ubiquitous and indispensable element in the human body, which directly affects the human metabolism and physiological activities. Hence, Fe-based MOFs have attracted an enormous amount of attention in the field of drug therapy.

**Figure 5:**
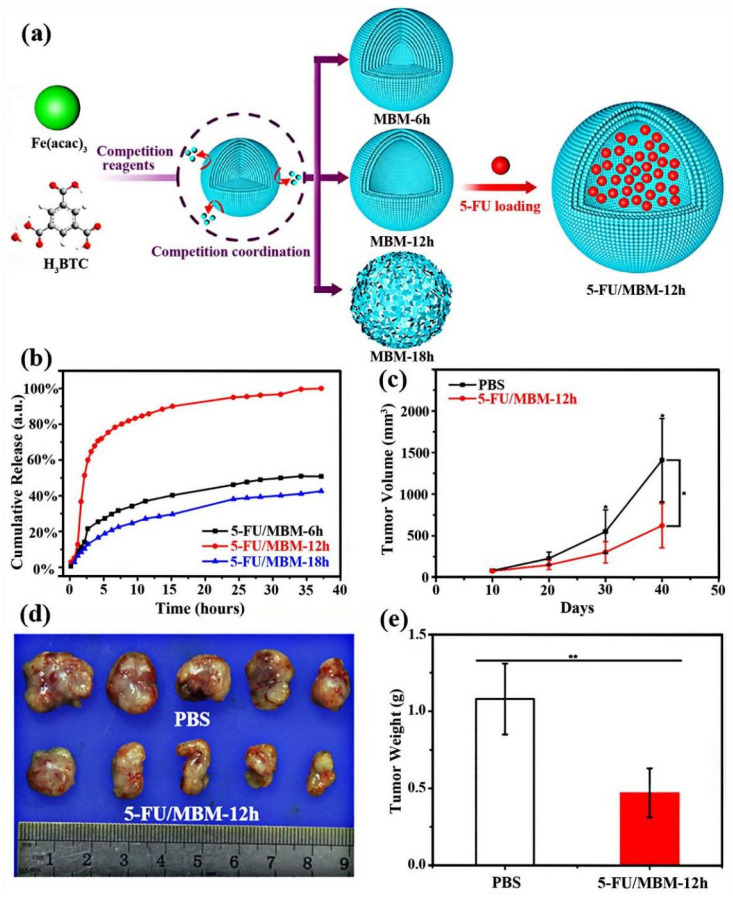
(**a**) The synthetic process of 5-FU/MBM-12 h. (**b**–**e**) Drug release and tumor treatment. Reproduced with permission from Ref. [75], copyright (2021) the American Chemical Society. (* *p* < 0.05; ** *p* < 0.01).

A typical work is reported by R. Cui and cooperators in 2021 (entry 8, Table 1) [75]. In this work, multifunctional Fe-based MOFs with a large pore size of 100−200 nm were successfully prepared by introducing polyoxometalates and sulfides as competing reagents during the in situ coordination assembly of Fe cations and benzene tricarboxylic acid (H_3_BTC) linkers at different reaction times (Figure 5a). The morphology, size, and microstructure of the Fe-MOF-based microcapsules (MBMs) were characterized in detail by SEM and TEM images. MBM-12 h has uniform approximate spherical shape with a particle diameter from 200 to 300 nm. In particular, MBM-12 h has a large hollow cavity (~200 nm) with a shell thickness of approximately 50 nm. The TEM−EDX showed that the elemental mapping of C, N, O, S, Mo, and Fe is dispersed homogeneously in MBM-12 h. The prepared MBM-12 h has a high stability and a large surface area, so it exhibits a maximum loading efficiency of 5-FU as high as 77%. The 5-FU release profiles of these 5-FU/MBM composites showed similar drug-release trends in PBS at a pH value of 7.4 at 37 °C (Figure 5b). The release rate of the 5-FU before the first 5 h was significantly higher than that after 5 h because the drug release from the outside surface and the shallow layer of the nanoscale MBM vehicles are easier than the drug-release process from the internal pore or cavity of porous nanocarriers. In comparison to the total drug-released amount of 5-FU/MBM-12 h, the released 5-FU amounts of 5-FU/MBM-6 h and -18 h were 62.9 and 52.7%, respectively, at the release time of 37 h, which confirms the optical-drug-release performance of 5-FU/MBM-12 h and the structure–property relationship during the drug-release process. Then, the 5-FU/MBM-12 h samples were separately dispersed in a PBS and aqueous solution, which were further injected into nude mice bearing A2780 tumors. As illustrated in Figure 5c, the 5-FU/MBM-12 h exhibits a more significant inhibition toward the tumor growth in the aqueous solution than that in the PBS, mainly due to the higher 5-FU loading and release capacities. Compared with the size and weight of the tumor in the PBS after 40 days, the 5-FU/MBM-12 h-treated tumor was significantly smaller and lighter, thus proving the good tumor-suppressive ability of 5-FU/MBM-12 (Figure 5d,e). This work not only develops multifunctional Fe-based MOFs to effectively inhibit the tumor growth via highly efficient drug delivery/release, but also studies the significant relationship between the drug-loading/release properties and the microstructures of MOFs. It explores an available and potential approach to enhance the clinical treatment effect by regulating the structure and morphology of MOFs.

**Figure 6:**
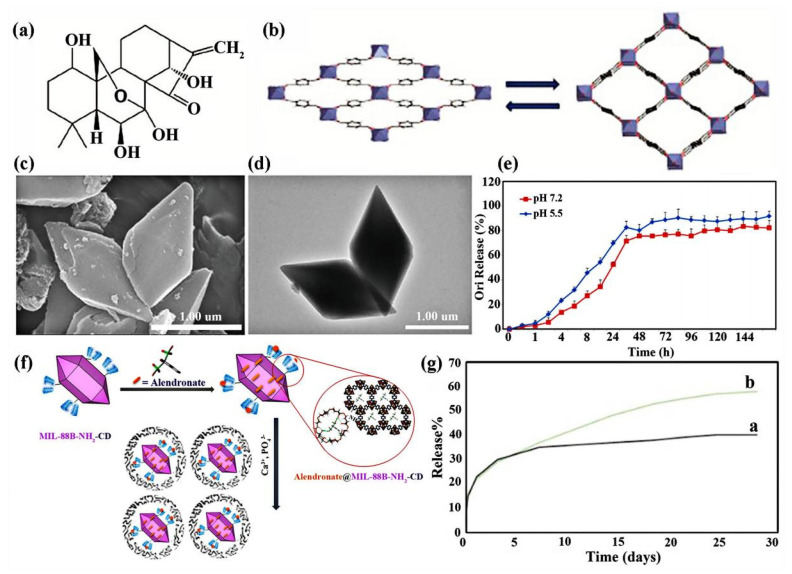
Structures of (**a**) Ori and (**b**) MIL-53(Fe). (**c**) SEM and (**d**) TEM images of Ori@MIL-53(Fe). (**e**) Ori release profiles of Ori@MIL-53(Fe). Reproduced with permission from Ref. [76], copyright (2018) MDPI, Basel, Switzerland. (**f**) Alen@Fe-MIL-88B-NH_2_-CD and (**g**) Alen release curves. Reproduced with permission from Ref. [121], copyright (2018) Springer.

In 2018, A. Pinna and coworkers prepared a hybrid material, namely PMP@MIL-88A, by growing MIL-88A(Fe) on the carboxyl-functionalized polymeric magnetic particles (PMPs) [121]. The as-synthesized PMP@MIL-88A NPs can efficiently deliver and release dopamine. The chemical stability of dopamine-loaded MIL-88A can be significantly improved due to the protective effect of MOFs. The PMP@MIL-88A nanocarriers can release dopamine into intracellular compartments and avoid side effects under extracellular conditions. Another MIL-53(Fe) was used as an available nanocarrier to load oridonin (Ori) (Figure 6a,b, entry 9, Table 1) [76]. The transmission electron microscope (TEM) and SEM images showed that Ori@MIL-53(Fe) retained the morphology and skeleton integrity of the original MIL-53(Fe) (Figure 6c,d). As illustrated in Figure 6e, the released Ori properties of Ori@MIL-53(Fe) are different at pH values of 5.5 and 7.2, illustrating that the drug-release capacity at a pH of 5.5 is higher than that at a pH of 7.2 within the same amount of time. These reports indicate that Fe-MOFs have good drug-loading capacities and controlled drug release to improve the therapeutic effect. Another interesting work is reported by A. Golmohamadpour and coauthors (entry 10, Table 1) [77]. They designed and prepared Fe-MIL-88B-NH_2_ with surface-functionalizedβ-cyclodextrines (β-CD) to obtain a drug nanocarrier (Fe-MIL-88B-NH_2_-CD). The as-synthesized Fe-MIL-88B-NH_2_-CD possessed a good capacity to efficiently load alendronate (Alen) (Figure 6f), which was further encapsulated by hydroxyapatite (Hap) to obtain Alen@Fe-MIL-88B-NH_2_-CD@Hap. The SEM images showed that Alen@Fe-MIL-88B-NH_2_-CD@Hap kept the main morphology of Fe-MIL-88B-NH_2_. The Alen release curves of Alen@Fe-MIL-88B-NH_2_-CD and Alen@Fe-MIL-88B-NH_2_-CD@Hap showed that the Hap coating can significantly enhance the Alen release amount after 5 days to improve the treatment effectiveness (Figure 6g). Both investigations illustrate that the drug-delivery and release properties of Fe-MOFs can be further improved through the efficient assembly with other functional materials. The particle size of the MOF carrier is still large enough to affect the drug load and release at the cellular level. Hence, the particle size had better control under 200 nm with a high dispersion to enhance the uptake by the cells.

**Figure 7:**
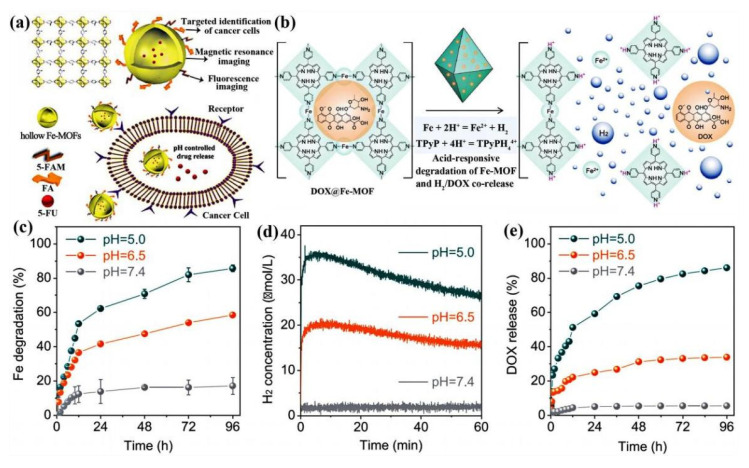
(**a**) Structure of Fe-MOF-5-NH_2_, hollow Fe-MOF-5-NH_2_-FA-5-FAM/5-FU and its pH-controlled drug delivery. Reproduced with permission from Ref. [78], copyright (2019) the Royal Society of Chemistry. (**b**) Acid-responsive degradation of Fe-MOF and H_2_/Dox release. (**c**) Degradability, (**d**) H_2_, and (**e**) Dox-release behaviors of Dox@Fe-MOF. Reproduced with permission from Ref. [79], copyright© 2022 The Authors. Advanced Science published by Wiley-VCH GmbH.

The design and development of stimuli-responsive drug nanocarriers with controlled-targeting delivery, good biocompatibility, and outstanding safety are extremely important in the clinical application. Some typical examples have been reported in recent years. In 2019, X. Gao and cooperators rationally prepared hollow Fe-MOF-5-NH_2_ NPs via an easy hydrothermal approach, which can be used as nanocarriers to load 5-FU with a maximum amount as high as 35% (entry 11, Table 1) [78]. The as-synthesized hollow Fe-MOF-5-NH_2_ NPs were further modified by folic acid (FA) and 5-Carboxylfluorescein (5-FAM) to generate Fe-MOF-5-NH_2_-FA-5-FAM/5-FU systems with a strong green fluorescence, good magnetic properties, as well as a pH-controlled 5-FU release (Figure 7a). The SEM and TEM images of the as-synthesized samples clearly displayed that Fe-MOF-5-NH_2_ NPs are hollow octahedral nanostructures with a particle size of ~200 nm. The rational integration of magnetic resonance and fluorescence imaging is significantly better than these individual components. Especially, the 5-FU release in vitro from Fe-MOF-5-NH_2_-FA-5-FAM/5-FU was studied in different PBS buffer solutions at pH values of 4, 5, 6, 7.4, and 8. The 5-FU release rates under these conditions were very fast in the first stage and became slow with the extension of time because the initially rapid release was caused by the 5-FU encapsulated in the external surface and the continuously slow release was mainly from the porous cavity of the MOFs. Then, Yao et al. successfully constructed an acid-degradable MOF nanocarrier with the production of H_2_ during the cancer-treatment process in 2022 (entry 12, Table 1) [79]. As illustrated in Figure 7b, FeCl_3_-MOF NPs were prepared via the coordination assembly of Fe(0) and 5,10,15,20-tetrakis(4-pyridyl)-21H,23H-porphine (TPyP), which can be disassembled under acid conditions to controllably release Dox drug molecules and generate H_2_ (Figure 7c–e). The acid-responsive skeleton destruction, H_2_ generation, and Dox-release properties of the nanoscale Dox@Fe-MOF carriers were studied and compared in different pH solutions. The degradation, H_2_ generation, and drug release of Dox@Fe-MOF gradually enhanced with the increasing acid concentration, implying the acid-responsive degradation of the MOFs with the drug release and H_2_ formation simultaneously. The MCF-7 and MCF-7/ADR tumor-bearing mice were used as research modes to evaluate the anticancer efficacy. The Fe-MOF NPs and free DOX can inhibit the growth of the MCF-7 tumor because of their corresponding chemotherapy and hydrogen therapy. On the other hand, Fe-MOF NPs and free DOX are not sensitive and effective toward MCF-7/ADR tumors. It was found that the Dox@Fe-MOF nanomedicine showed a more outstanding therapeutic outcome than that of the individual component toward both MCF-7 and MCF-7/ADR tumors by virtue of the synergistic therapeutic effect of the hydrogen therapy and chemotherapy of the Dox@Fe-MOF. More importantly, the dual-hydrogen-chemotherapy of the Dox@Fe-MOF with an excellent therapeutic effect does not result in a significant weight loss in both tumors. The results illustrate that Fe-MOF NPs provide a platform for drug carriers and hydrogen donors to synergistically treat tumors in clinical applications. Few MOF-based carriers have been reported so far, so it is a great challenge to prepare stimuli-responsive drug nanocarriers for improving targeting and synergistic therapies.

Some pH-responsive smart Fe-MOF nanocarriers have also been reported in controlled and targeted drug delivery/release [122,123,124,125]. By virtue of the nontoxic Fe element, large surface area, nanoscale crystal, and good designability of the Fe-MOFs, it is a greatly potential vehicle to efficiently load drugs and perform the targeted release for improving the effect of tumor treatments. Among the different metal-based MOFs, high-crystallinity porous Fe-MOFs are more difficult to obtain generally. Hence, it is still a great challenge to construct novel Fe-MOFs NPs with a low toxicity, high biocompatibility, a large porous structure, and a sensitive responsiveness for a high-drug-loading amount, controlled drug delivery, and an excellent synergistic treatment effect in clinical areas.

## 4. CD MOFs

Cyclodextrins (CDs) belong to a class of natural circular oligosaccharides via the α-1,4-glycosidic linkage. The three main constitutions of CD molecules are α, β, and γ-CDs, with different depths and diameters of the interior cavities, resulting in their wide utilization to immobilize different species via the host–guest interaction [126,127,128,129]. Meanwhile, the oxygen-containing functional groups of CDs provide functional sites to coordinate with metal ions to construct CD MOFs, which have many advantages such as large cavities, well-defined pores, nontoxicity, and a good biocompatibility. Hence, CD MOF nanocarriers are favored for drug loading and release in the therapeutic field. Recently, more attention has been focused on the development of nanoscale CD MOFs for controlled drug delivery and release [130,131,132,133,134,135,136,137,138,139,140].

**Figure 8:**
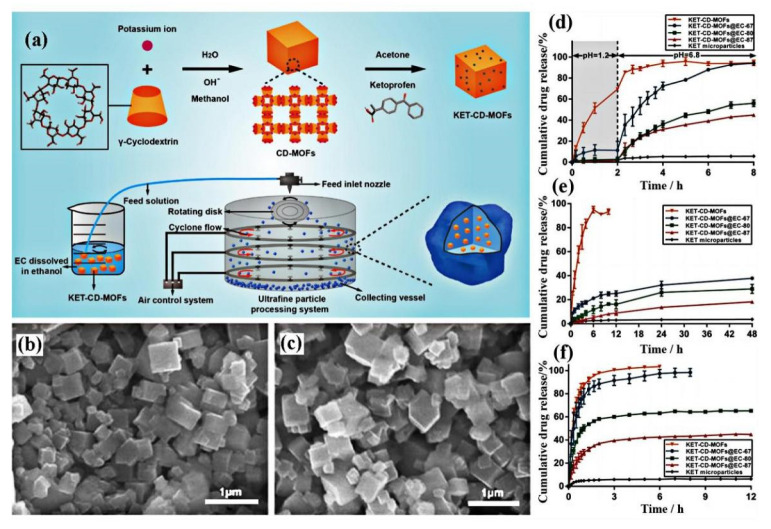
(**a**) Preparation, (**b**,**c**) characterization, and (**d**–**f**) drug delivery of CD-MOFs and KET-CD-MOFs. Reproduced with permission from Ref. [80], copyright (2019) the American Chemical Society.

Some representative examples from the last five years are further highlighted and discussed in this review. CD-MOFs are constructed via the coordination assembly of γ-CD and K^+^, which is a good nanocarrier to load ketoprofen (KET), to obtain KET-CD-MOFs (entry 13, Table 1) [80]. In addition, the as-synthesized KET-CD-MOFs can be further coated with ethylcellulose (EC) via the ultrafine particle processing system to generate composite microparticles (Figure 8a). The morphology and particle size of CD-MOFs and KET-CD-MOFs are observed by SEM images (Figure 8b,c), demonstrating that they have a similar cubic morphology with a particle size of ~400 nm. The XRD patterns of both CD-MOFs and KET-CD-MOFs are also similar to each other, confirming the crystal structure of CD-MOFs during the KET-loading process. The XRD characteristic signals of KET disappear in KET-CD-MOFs because of the amorphous or molecular state of KET encapsulated in the pores of the host MOFs. N_2_ adsorption/desorption isotherms show that the Brunauer−Emmett−Teller (BET) surface area decreases from 816 m^2^ g^−1^ in CD-MOFs to 20 m^2^ g^−1^ in KET-CD-MOFs because the pores of the MOFs are mostly filled by the guest KET molecules. KET-CD-MOFs and microparticles are immersed in simulated gastric fluid (SGF) and intestinal fluid (SIF) to study the drug release properties. As depicted in Figure 8d−f, the drug-release properties can be well-controlled by regulating the assembly ratio of the EC and CD-MOFs. Moreover, the drug-release amounts of the composite microparticles are lower than that of the KET-CD-MOF, which illustrates that the majority of the KET-CD-MOFs are encapsulated into the microparticles. This work provides a mild one-step-encapsulation approach to introduce the protective polymers over the CD-MOFs for a controlled drug release and enhanced biocompatibility. Nevertheless, the drug-loading amount of the CD-MOFs is still low and the drug-release rate is not smooth enough. If CD-MOFs can enhance the drug load and keep a relatively uniform drug release, the patient or user will only take the drug once to ensure a long-term drug-release treatment.

**Figure 9:**
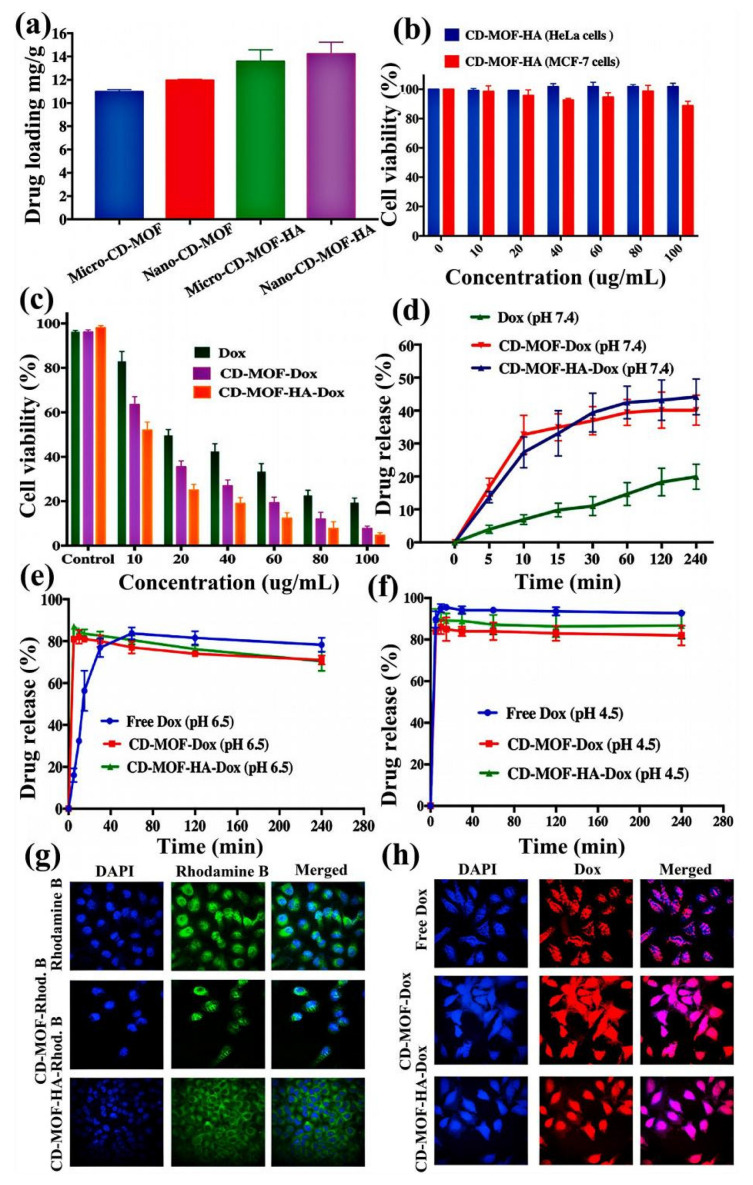
The bioapplication of CD-MOF-based carriers. Reproduced with permission from Ref. [81], copyright (2022) Elsevier.

Another acid-responsive biofunctionalized γ-CD-based MOF is successfully prepared via a simple postmodification method, which is a good nanocarrier to load Dox and a pH-responsive drug release with a high cellular uptake (entry 14, Table 1) [81]. Hyaluronic acid (HA) is easily interacted with the overexpressed cancer CD44 receptor, so HA is used to modify the CD-MOFs to obtain CD-MOF-HA. Meanwhile, the particle size of the CD-MOFs can be well-adjusted from the micro- and nanometer scales. SEM images show that the cubic CD-MOF is well-kept after the crosslinking procedure, but the structure and crystallinity of the CD-MOFs has a certain amount of damage after the surface-modification process. The XRD peaks of the HA-modified CD-MOFs become weaker and wider than that of the nano-CD-MOF-HA, further suggesting the partial structural destruction of the CD-MOFs after modification with HA. As illustrated in Figure 9a, the nano-CD-MOF-HA has the best drug-loading capacity among all the used CD-MOF-based carriers. The average Dox-loading amount of the CD-MOF-HA is approximately 4.8% higher than those of the CD-MOFs. The biocompatibility of the CD-MOF-HA is investigated in both MCF-7 and HeLa cancer cells. The high cell viability of both cells at different amounts of the CD-MOF-HA strongly proves the biofriendly nature of the CD-MOF-HA nanocarriers (Figure 9b). Compared with the cell viability of the free drug and CD-MOF-Dox, the CD-MOF-HA-Dox exhibits a higher toxicity toward HeLa cells (Figure 9c). The CD-MOF-Dox and CD-MOF-HA-Dox show similar drug-release behaviors, which are higher than that of the free Dox at a pH of 7.4 (Figure 9d), because the CD-MOFs have some characteristics, such as a hydrophilic nature, an easy-decomposition nature, and a high surface area, to improve the drug-release amount. The pH values of the tumor tissue and the endosomal environment are 6.5 and 4.5, respectively. The drug-delivery profile of the CD-MOF-HA is up to six times higher than that of free Dox; Dox can be totally released within only 5 min (Figure 9e). In the endosome microenvironment, the CD-MOF-HA-Dox and free Dox exhibit similar burst release profiles (Figure 9f). As a result, the CD-MOF-HA nanocarrier can effectively release Dox in the acid condition. Moreover, the CD-MOF-HA with rhodamine B reveals an obvious green fluorescence near the cell nucleus, suggesting that the CD-MOF-HA binds to HeLa cells (Figure 9g). It confirms that the binding interaction of HA in CD-MOF-HA can keep well after attaching on CD-MOFs. However, free Dox is distributed irregularly in the cells. The fluorescence intensity of the CD-MOF-HA is stronger in HeLa cells than that of free Dox, indicating the good cellular penetration of the Dox in cells (Figure 9h). This work develops a useful approach to synthesize biofunctionalized CD-MOFs as drug nanocarriers for targeted drug delivery. However, the drug-release rate is too fast to satisfy the actual demand for a sustained drug release and to reduce the targeting of drug delivery in cancer cells. Therefore, it is important to properly improve the stability of nanocarriers to prolong the drug-release time.

**Figure 10:**
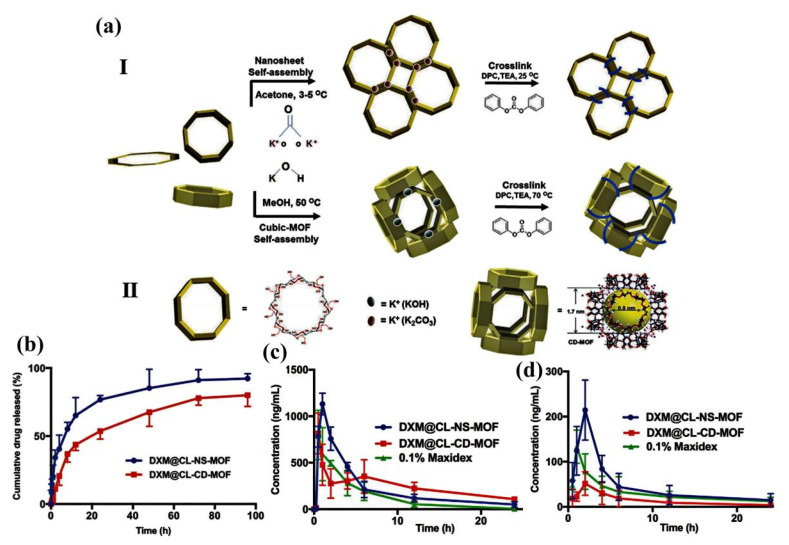
(**a**) NS- and CD-MOFs with an effective carbonyl crosslink. (**b**) Drug-release curves of DXM@CL-CD-MOF and DXM@CL-NS-MOF in a simulated tear fluid. DXM concentration–time profiles of DXM@CL-CD-MOF and DXM@CL-NS-MOF in (**c**) tear fluid and (**d**) aqueous humor. Reproduced with permission from Ref. [82] copyright (2020) Wiley-VCH.

The morphology and size of the CD-MOF-based nanocarriers are still very important in drug delivery and release. As reported by M. G. Bello and coauthors in 2020, 2D nanosheets (NSs) of γ-CD-based MOFs (NS-MOFs) and a cubic-MOF were prepared using a feasible one-pot reaction of K_2_CO or KOH as metal sources under different conditions (entry 15, Table 1) [82]. It is disappointing that both as-synthesized CD-MOFs did not have a good stability in water, so the highly stable crosslinked cubic CD-MOF (CL-CD-MOF) and NS-MOF (CL-NS-MOF) were rationally fabricated via a polymerization process, as shown in Figure 10a. Both samples were used to load dexamethasone (DXM), with a DXM-loading percent of 6.5% in CL-NS-MOF and 6% in CL-CD-MOF, respectively. The SEM images illustrate that the morphology of both particles can keep well after loading DXM. The DXM cumulative release formed DXM@CL-NS-MOF and DXM@CL-CD-MOF and were performed under simulated physiological conditions (Figure 10b). Both composites showed rapid and slow drug-release processes. The DXM@CL-NS-MOF showed more DXM release (73%) than the DXM@CL-CD-MOF (55%) in the first 24 h, which was mainly caused by the easier drug release of the nanosheet DXM@CL-NS-MOF than that of the cubic-shaped carrier. Finally, the DXM@CL-NS-MOF could almost entirely release DXM after 96 h in this medium. The tear-fluid-elimination-kinetics analysis and drug concentrations in the aqueous humor of DXM@CL-CD-MOF, DXM@CL-NS-MOF, and Maxidex were investigated and compared with each other under the same conditions (Figure 10c,d). The whole-concentration time data manifested that the DXM@CL-NS-MOF nanosheet carriers can effectively promote drug dissolution. This work illustrates that the size and morphology of the MOF-based nanocarriers may directly affect the drug-release performance in the clinical treatment field.

**Figure 11:**
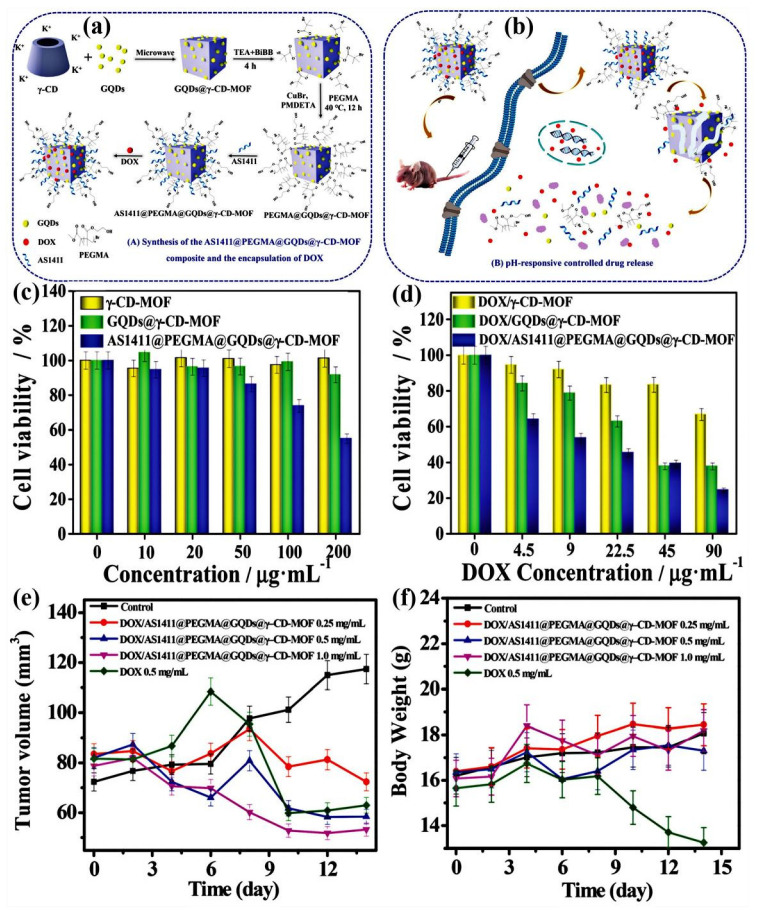
(**a**) Preparation of Dox/AS1411@PEGMA@GQDs@γ-CD-MOF and (**b**) its pH-responsive Dox release. (**c**,**d**) Cell viability of different carriers. (**e**) Tumor volume growth and (**f**) body weights of mice treated by drug-delivery systems. Reproduced with permission from Ref. [83], copyright (2019) the Royal Society of Chemistry.

To achieve a more effective targeted drug delivery, Q. Jia and coauthors designed and prepared a targeted-drug-delivery vehicle (entry 16, Table 1) [83]. The nanocarriers could be rationally constructed by γ-CD-MOF and graphene quantum dots (GQDs) (namely, GQDs@γ-CD-MOF), which was further modified by poly(ethylene glycol) methacrylate (PEGMA) via surface-initiated atom transfer radical polymerization (SI-ATRP) to obtain the hybrid material (namely, PEGMA@GQDs@γ-CD-MOF) (Figure 11a). Especially, the nanoscale AS1411@PEGMA@GQDs@γ-CD-MOF carrier exhibited a high-Dox-loading amount, a pH-controlled drug release, and a good biocompatibility (Figure 11b). As displayed in Figure 11c, the γ-CD-MOF and GQDs@γ-CD-MOF have a high biocompatibility toward MCF-7 cells even at the concentration of 200 μg mL^−1^, confirming their nontoxicity and biosecurity in the drug delivery. The PEGMA-modified GQDs@γ-CD-MOF can adsorb the AS1411 aptamer to enhance the cytotoxicity to 54.9% at 200 μg mL^−1^, which is probably ascribed to the anticancer agent AS1411 for cell death. On the other hand, the cell-inhibition rates of Dox-loaded nanocarriers show the positive correlation with Dox-release rates (Figure 11d). In comparation with all drug-delivery systems, DOX/AS1411@PEGMA@GQDs@γ-CD-MOF exhibited the highest cytotoxicity toward MCF-7 cells due to the synergistic effect of the controlled Dox release inside the cancer cells under a low pH condition and the specific binding of AS1411 toward MCF-7 cells. As seen in Figure 11e, the tumor volume was measured every 2 days after using different amounts of DOX/AS1411@PEGMA@GQD@γ-CD-MOF. It was found that the tumor growth can be restrained effectively because of the targeted effect of AS1411 in the DOX/AS1411@PEGMA@GQDs@γ-CD-MOF system. Moreover, almost no weight loss for the mice was found after treatment with DOX/AS1411@PEGMA@GQDs@γ-CD-MOF, but the treatment with free Dox exhibited a significant weight loss, suggesting the good biocompatibility of the DOX/AS1411@PEGMA@GQDs@γ-CD-MOF. Hence, this work develops a unique nanocomposite with the advantages of each component, including the good solvability and biocompatibility of γ-CD-MOF, the highly fluorescent GQD, the targeted AS1411, and the pH-responsive PEGMA. It opens up a useful approach to realize efficient tumor-targeted drug delivery in vitro and in vivo to further enhance the tumor therapeutic effect.

Hence, CD-MOFs and their derivates, as potential drug carriers, have many special advantages to improve the clinical therapeutic effect. However, it is still a great challenge to develop more CD-MOFs with large pores for loading drug macromolecules. If such macroporous CD-MOFs can be prepared in large quantities, it will promote the rapid development and application of drug-delivery materials. Meanwhile, the particle size and morphology of CD-MOF-based carriers require more attention to achieve highly efficient drug delivery and cell uptake. Moreover, the introduction of targeted molecules and aptamers in the CD-MOF-based drug-delivery systems is an available approach to enhance the targeted drug delivery to cancer cells. According to these considerations, the rapid development of CD-MOF-based drug-delivery systems have great application prospects in the field of basic research and clinical treatment.

## 5. Other MOFs

In addition to the above materials, other MOFs have been designed and prepared successfully as drug carriers to improve the therapeutic efficacy [141,142,143,144,145,146,147,148,149,150,151,152,153,154,155,156,157,158]. Similar with the Fe-MOFs, UiOs, and CD-MOFs, other MOFs are constructed by metal ions/clusters (Zn^2+^, Cu^2+^, and Bi^3+^) and bridging linkers. Compared with the above-mentioned MOFs, other MOFs have relatively high toxic metals, such as Cu^2+^, Ni^2+^, and so on, resulting in their poor biocompatibility and safety in the in vivo experiments or clinical treatments. Therefore, more attention has been focused on the high-drug-loading amount and low-dose injection into cells, resulting in fewer carrier materials with as high a drug release as possible. Some representative studies will be highlighted and discussed in the field of drug delivery.

**Figure 12:**
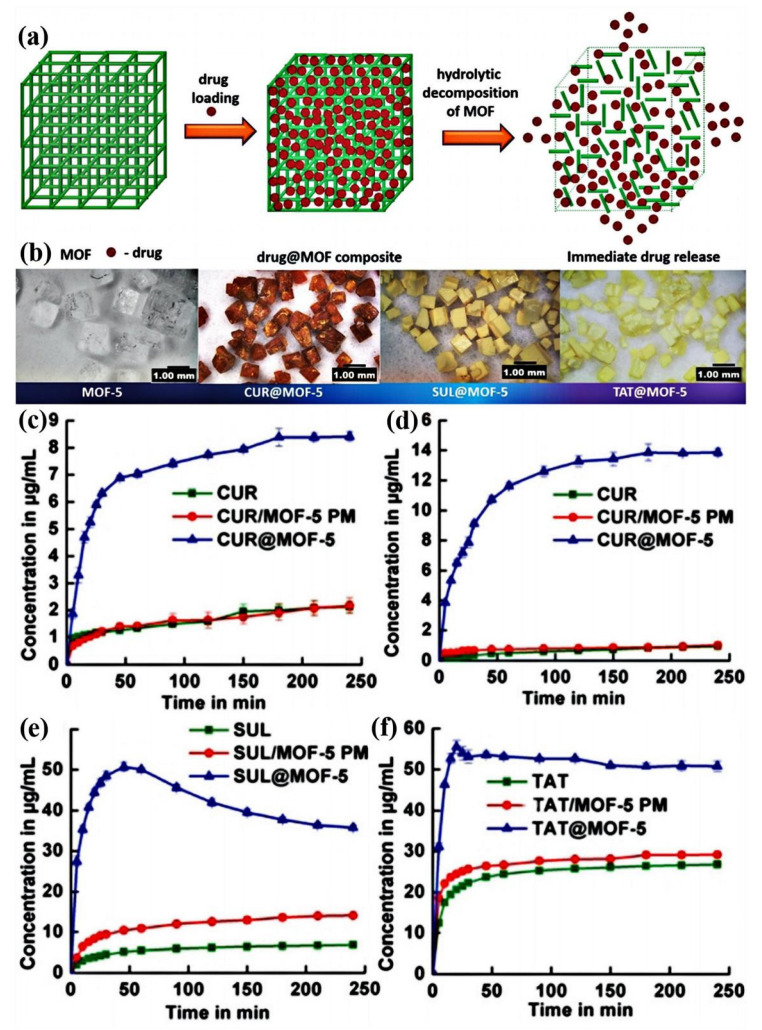
(**a**) The formation of drug@MOF and drug release. (**b**) Optical images of MOF-5 and drug@MOF-5. Dissolution profiles: CUR, CUR@MOF-5, and CUR/MOF-5 PM in (**c**) SG and (**d**) PBS; (**e**) SUL, SUL@MOF-5, and SUL/MOF-5 PM in SG; (**f**) TAT, TAT@MOF-5, and TAT/MOF-5 PM in PBS. Reproduced with permission from Ref. [84], copyright (2019) Wiley-VCH.

Drug molecules can be encapsulated into water-sensitive MOFs, leading to the rapid release of amorphous drugs in the water systems via the hydrolytic decomposition of MOFs, and the prevention of pharmaceutical crystallization, thus enhancing the saturated solubility of the drugs (Figure 12a). In order to verify this hypothesis, K. Suresh and A. J. Matzger selected three poorly water-soluble drugs, including curcumin (CUR), sulindac (SUL), and triamterene (TAT), to investigate the loading and release properties of activated MOF-5 (entry 17, Table 1) [84]. The drug@MOF-5 can be collected after washing with fresh solvents to remove the residual drug on the MOF’s surface. The colors of the drug@MOF-5 composites are obviously different from MOF-5, including brick red for CUR@MOF-5, yellow for SUL@MOF-5, and light yellow for TAT@MOF-5 (Figure 12b). In addition, the XRD patterns of the drug@MOF-5 composites illustrate that the structure of MOF-5 can be kept well during the encapsulating process of the drug. All characteristic peaks of the drug were dispersed in the drug@MOF-5 composites because the small pores of the MOFs can confine the drug molecules to impede the formation of the crystalline form. As seen in Figure 12c−f, the free drug, drug@MOF-5 composites, and physical mixtures (PMs) are dissolved in simulated gastric (SG) and PBS media to investigate the drug-dissolution rate and supersaturation generation. The results illustrated that the drug@MOF-5 can achieve a rapid dissolution and improve the supersaturation of the drug, which are significantly superior to those of the free drug and the drug/MOF-5 physical mixtures (PMs) under the same medium. This work develops a novel method to improve the solubility of the drug with poor water solubility and promotes drug release using water-sensitive MOF carriers. Water-sensitive MOFs are potential drug-carrier capsules to load poorly soluble drugs, resulting in the formation of host–guest drug-delivery systems for improving the drug solubility and increasing the clinical treatment effect.

**Figure 13:**
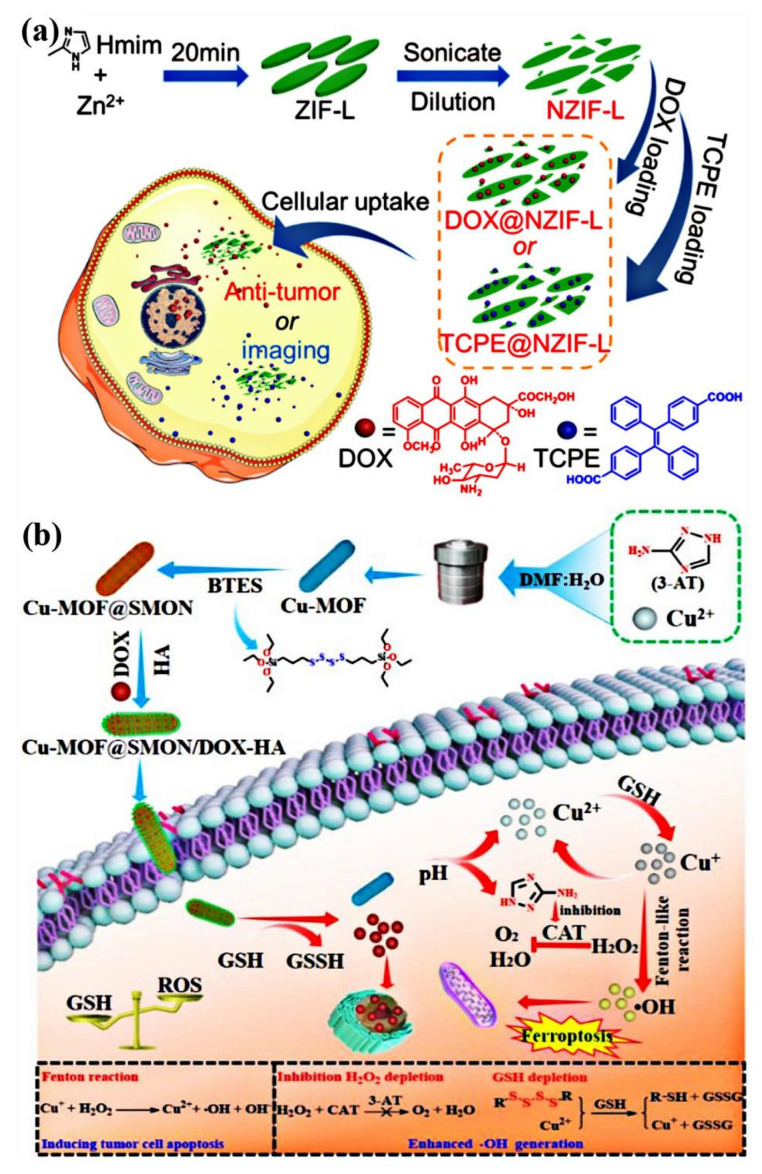
(**a**) The construction of NZIF-L and its application in Dox delivery and imaging. Reproduced with permission from Ref. [85], copyright (2019) Royal Wiley-VCH. (**b**) The synthetic process of Cu-MOF@SMON/DOX-HA and its biological application. Reproduced with permission from Ref. [86], copyright (2022) the Royal Society of Chemistry.

Multiresponsive functional nanocarriers still need further investigation to enhance the therapeutic effect. For example, J. Dong and coworkers successfully constructed a pH- and GSH-responsive core-shell hybrid material as a chemodynamic therapy (CDT)/chemotherapy (CT) dual-therapeutic agent (entry 18, Table 1) [85]. As seen in Figure 13a, the Cu-MOF core is synthesized via the coordination assembly of 3-amino-1,2,4-triazole (3-AT) and Cu^2+^, which is further covered by a porous shell using bis[3-(triethoxysilyl)propyl]tetrasulfide. The prepared Cu-MOF@SMON was used as a carrier to load Dox to generate Cu-MOF@SMON/DOX, which can be wrapped by HA via the electrostatic interaction to improve the targeting performance toward HepG2 cells. By virtue of the GSH-responsive tetrasulfide bond and acid-responsive decomposition of the MOFs, the Cu-MOF@SMON/DOX-HA is considered as a pH/GSH dual-responsive drug-delivery system. It is worth noting that the free Cu^2+^ can generate ·OH by catalyzing H_2_O_2_ and the 3-AT can restrain the catalase (CAT) enzyme activity. This proposed strategy can efficiently fabricate core–shell organic mesoporous materials@microporous MOFs to enhance the loading amount of large drug molecules and the anticancer efficacy. Hence, this work provides a novel and interesting idea for multiresponsive core–shell MOF-based hybrid materials in clinical treatments. Moreover, as the size and morphology of the MOFs provide further benefit for the drug delivery and release, Y. Song and et al. designed and prepared a leaf-shaped zeolitic imidazolate framework (namely, ZIF-L) via a low molar ratio of 2-methylimidazole (Hmim) and Zn^2+^ in an aqueous system for 20 min (Figure 13b, entry 19, Table 1) [86]. The as-synthesized ZIF-L can be used as an outstanding cargo carrier to load 4,4’-(1,2-diphenylvinyl)-1,2-di-(phenylcarboxylic acid) (TCPE) and Dox. The formed TCPE@NZIF-L and DOX@NZIF-L exhibited their potential applications in therapy and cell imaging toward RAW264.7 cells. This study highlights that nanoscale MOF sheets are potential carriers toward therapeutic and imaging agents.

More importantly, Zn-based zeolitic imidazolate frameworks (ZIFs) have been widely used as nanocarriers for controlled drug delivery and release because of the good biocompatibility, morphological controllability, low toxicity, and small particle size of the ZIFs [159,160,161,162,163,164,165,166,167,168,169,170,171,172,173]. Meanwhile, the ZIFs are easily assembled with other functional materials to enhance the drug-treatment effect. Among all the reported ZIFs, ZIF-8, constructed using Zn^2+^ and 2-methylimidazolate (2-MeIM), is considered as the most frequently used nanocarrier to load drugs in the field of drug therapy because ZIF-8 has excellent biocompatibility, nontoxicity, a low cost, well-defined pores, and an easy preparation. Some typical examples are highlighted and discussed in this part. Notably, ZIF-8 is easily broken under weak acid conditions, which can be utilized in the targeted drug release in cancer cells in the acid environment.

**Figure 14:**
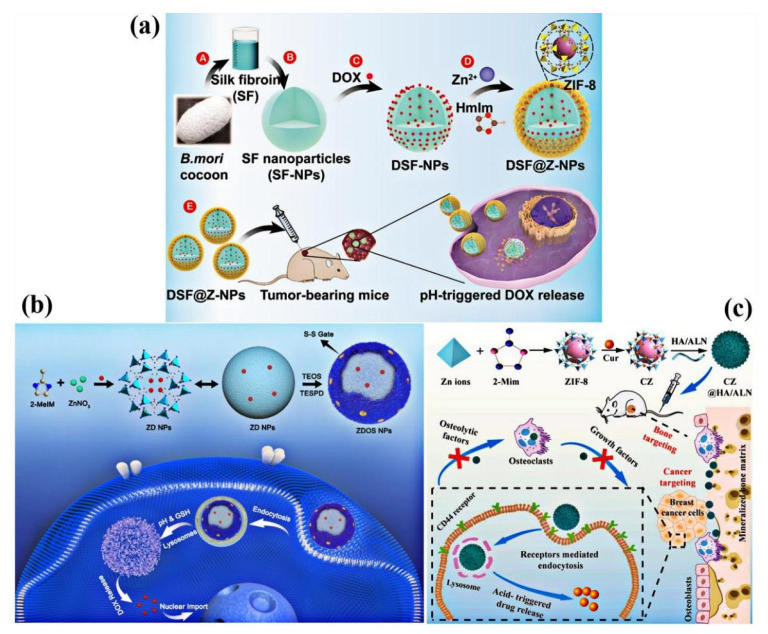
Schematic diagrams of the preparation of (**a**) DSF@Z-NPs, (**b**) ZIF-8@DOX@organosilica, and (**c**) CZ@HA/ALN for drug delivery. Reproduced with permission from Ref. [87], copyright (2021) the American Chemical Society, Ref. [88], copyright (2019) the American Chemical Society, and Ref. [89], copyright (2022) Elsevier.

As illustrated in Figure 14a, silk fibroin (SF) biomacromolecules were assembled to form nanoparticles (SF-NPs) to load the Dox antitumor drug. The resultant DSF-NPs were further used as the nucleation to grow ZIF-8 shells, leading to the formation of core–shell DSF@Z-NPs. By virtue of the selective dissolution of ZIF-8 under an acidic pH and with a high stability under neutral conditions, the prepared DSF@Z-NPs can be efficiently uptaken by cancer cells and selectively release Dox in the acidic intracellular environment of breast cancer cells. Hence, this carrier can more effectively inhibit tumor growth and reduce damage to normal cells (entry 20, Table 1) [87]. Another dual stimulus-response carrier was successfully constructed by the integration of ZIF-8 cores and organosilica shells. As shown in Figure 14b, the anticancer drug Dox was firstly loaded in ZIF-8 via an in situ encapsulation approach. The as-synthesized ZIF-8@Dox (namely, ZD NPs) was further coated using biodegradable organosilica with disulfide linkages to obtain core–shell nanocarrires (ZDOS NPs), which not only exhibited a high-Dox-loading amount of 41.2% but also possessed significant pH- and glutathione (GSH)-sensitive drug-release behaviors. This vehicle could keep its structure in the physiological environment and concentrate at the tumors, but the Dox could be controlled-released into cancer cells because of the unstable disulfide linkages in the existence of endogenous GSH and the easy dissociation of ZIF-8 under the acidic cancer environment. This work develops a dual-responsive ZIF-8-based hybrid material to deliver drugs into target cancer cells via the utilization of the structural characteristics and stability of different components (entry 21, Table 1) [88]. Remarkably, the collaborative treatment of multiple diseases is still very important in healthcare, so Y. Shen and Y. Lv developed a dual-targeted ZIF-8 carrier to simultaneously deliver a curcumin (Cur) and NF-κB inhibitor for bone and tumor treatments (Figure 14c). Cur could be trapped into porous ZIF-8 (namely, CZ), which was further modified by the tumor- and bone-targeting ligands of hyaluronic acid (HA) and alendronate (ALN) (namely, CZ@HA/ALN). HA and ALN could greatly improve the accumulation of NPs in the tumor bone metastasis after intravenous injection. Subsequently, the pH-responsive ZIF-8 was decomposed to release Cur for medical treatment. The dual-targeted drug-delivery system opens up an available approach to achieve the synergistic therapy for suppressing tumor progression and antibone resorption (entry 22, Table 1) [89]. These investigations confirm ZIFs are a class of potential nanocarriers for controlled drug delivery and release. Hence, more attention should be focused on efficient ZIF-based hybrid materials for enhancing the therapeutic effect and expanding their applications in other disease fields.

According to these reports, nanometer-sized porous MOFs and their hybrid materials with a thin layer morphology and sensitive species have a certain potential application value for drug delivery and controlled release in clinical settings. Meanwhile, the solubility and saturation of drugs in water systems and biological fluids can be obviously improved via immobilization in the pores of the MOFs, resulting in the dissolution, utilization, and absorption of drugs in the human body.

## 6. Challenges and Opportunities

Recently, nanoscale MOFs for on-demand drug release have received lots of attention due to their great potential in applications for cancer therapy. Meanwhile, MOFs can be used as a main component of drug-delivery systems to assemble with other functional materials, such as polymeric coatings, to enhance the treatment effect. Although the fast progress has been widely focused on the development of MOF-based drug carriers, there are some tricky issues that still need to be solved before its employment in practical applications.

(1) The rational preparation of novel porous MOFs is one of the key areas. Especially, organic linkers play an important role in the construction of functional MOFs, such as calixarenes, rotaxanes, catenanes, etc. These bridging ligands can coordinate with different metal ions/clusters to obtain porous MOFs, which may be used as potential carriers for drug delivery and release. The porous skeleton can enhance the drug-loading capacity, especially large porous MOFs. Meanwhile, the nanoscale and lamellar MOFs are good for internal circulation, cell absorption, and drug release.

(2) Biocompatibility is necessary for MOF-based drug carriers. The N, O, and S heteroatoms of drug molecules can be used to coordinate with nontoxic nodes to form porous MOFs, such as medical MOFs constructed from bioactive curcumin. It can not only ensure good biocompatibility, but also provide a feasible path for the simultaneous delivery of dual drugs.

(3) Some functional species can be assembled with MOFs to enhance the drug-loading capacity, pH- or thermal-responsive drug release, and good targeting effect. The target species can enhance the therapeutic efficacy and minimize concomitant side effects. The design and synthesis of stimuli-responsive biocompatible MOF-based drug-delivery systems still requires more effort. The low toxicity and good colloidal stability of nanoscale MOFs should attract lots of attention.

(4) It is important to develop effective approaches and technologies to combine MOFs and functional species, such as coordination and covalent bonds. Effective means are not only conducive to the preparation of hybrid materials but can also preserve their inherent advantages to achieve a synergistic effect.

## 7. Conclusions

In this review, we summarized the recent development of MOF-based nanocarriers for drug delivery and release in the last five years, including UiOs, Fe-MOFs, CD MOFs, and other representative MOFs. This review confirms that MOFs provide a potential platform for drug delivery and controlled release for enhancing therapeutic efficacy. More attention has been focused on the development of MOF-based drug-delivery systems in the clinical area, but the drug-delivery and release properties of MOFs are still far from reaching the requirements of practical application. In conclusion, the development of MOF-based drug-delivery systems with a low toxicity, biodegradability, and a high therapeutic efficacy is a promising research direction, but there is still a long way to go before their clinical application.

## Figures and Tables

**Table 1:** Some representative MOF-based nanocarriers for drug delivery.

Entry	MOF	Metal	Organic Ligand	Drug	Ref.
1	Zr-MOFs (L1–6)	Zr	2,6-napthalenedicarboxylate, 4,4′-biphenyldicarboxylate, 1,4-benzenedicarboxylate and its derivatives (-Br, -NO_2_, and -NH_2_)	5-fluorouracil	[68]
2	UiO-66-NH_2_	Zr	2-aminoterephthalic acid	ibuprofen	[69]
3	UiO-67-NH_2_	Zr	2-amino-4,4′-biphenyl dicarboxylic acid	camptothecin	[70]
4	NH_2_-UiO-66	Zr	2-aminoterephthalic acid	doxorubicin	[71]
5	MOF-808	Zr	1,3,5-benzenetricarboxylate	floxuridine and carboplatin	[72]
6	UiO-66	Zr	1,4-benzenedicarboxylate	camptothecin and doxorubicin	[73]
7	UiO-66-NH_2_	Zr	2-aminoterephthalic acid	methotrexate	[74]
8	MBM-12 h	Fe	1,3,5-benzenetricarboxylate	5-fluorouracil	[75]
9	MIL-88A(Fe)	Fe	1,4-benzendicarboxylate	oridonin	[76]
10	Fe-MIL-88B	Fe	2-aminoterephthalic acid	alendronate	[77]
11	Fe-MOF-5-NH_2_	Fe	2-aminoterephthalic acid	5-fluorouracil	[78]
12	FeCl_3_-MOF	Fe	5,10,15,20-tetrakis(4-pyridyl)-21*H*,23*H*-porphine	doxorubicin	[79]
13	CD-MOFs	K	γ-CD	ketoprofen	[80]
14	γ-CD-MOF	K	γ-CD	doxorubicin	[81]
15	γ-CD-MOF	K	γ-CD	dexamethasone	[82]
16	γ-CD-MOF	K	γ-CD	doxorubicin	[83]
17	MOF	Zn	1,4-benzenedicarboxylate	curcumin, sulindac, and triamterene	[84]
18	ZIF-L	Zn	2-methylimidazole	doxorubicin	[85]
19	Cu-MOF	Cu	3-amino-1,2,4-triazole	doxorubicin	[86]
20	ZIF-8	Zn	2-methylimidazole	doxorubicin	[87]
21	ZIF-8	Zn	2-methylimidazole	doxorubicin	[88]
22	ZIF-8	Zn	2-methylimidazole	curcumin	[89]

## Data Availability

The study did not report any data.
